# Real-World Outcomes of First-Line FOLFIRI Plus Bevacizumab with Irinotecan Dose Escalation versus FOLFOXIRI Plus Bevacizumab in *BRAF^V600E^*-Mutant Metastatic Colorectal Cancer: The Preliminary Data from a Single-Center Observational Study

**DOI:** 10.3390/medicina59122108

**Published:** 2023-12-01

**Authors:** Hsiang-Lin Tsai, Ching-Wen Huang, Yen-Cheng Chen, Wei-Chih Su, Tsung-Kun Chang, Po-Jung Chen, Ching-Chun Li, Yu-Tang Chang, Jaw-Yuan Wang

**Affiliations:** 1 Division of Colorectal Surgery, Department of Surgery, Kaohsiung Medical University Hospital, Kaohsiung Medical University, Kaohsiung 80708, Taiwan; chunpin870132@yahoo.com.tw (H.-L.T.); baseball5824@yahoo.com.tw (C.-W.H.); googoogi05@gmail.com (Y.-C.C.); lake0126@yahoo.com.tw (W.-C.S.); tsungjunchang@gmail.com (T.-K.C.); glaudiotennis@gmail.com (P.-J.C.); dobird05@yahoo.com.tw (C.-C.L.); 890300@ms.kmuh.org.tw (Y.-T.C.); 2 Department of Surgery, Faculty of Medicine, College of Medicine, Kaohsiung Medical University, Kaohsiung 80708, Taiwan; 3 Graduate Institute of Clinical Medicine, College of Medicine, Kaohsiung Medical University, Kaohsiung 80708, Taiwan; 4 Department of Surgery, Faculty of Post-Baccalaureate Medicine, College of Medicine, Kaohsiung Medical University, Kaohsiung 80708, Taiwan; 5 Division of Pediatric Surgery, Department of Surgery, Kaohsiung Medical University Hospital, Kaohsiung Medical University, Kaohsiung 80708, Taiwan; 6 Graduate Institute of Medicine, College of Medicine, Kaohsiung Medical University, Kaohsiung 80708, Taiwan; 7 Center for Cancer Research, Kaohsiung Medical University, Kaohsiung 80708, Taiwan

**Keywords:** *BRAF^V600E^* mutation, metastatic colorectal cancer, *UGT1A1* polymorphism, irinotecan dose escalation, progression-free survival, overall survival

## Abstract

*Background and Objectives*: Approximately 5–10% of all patients with metastatic colorectal cancer (mCRC) harbor a *BRAF^V600E^* mutation. These patients exhibit distinct metastatic patterns, poor prognosis, and heterogenous survival outcomes. The findings from the TRIBE study indicated that the administration of FOLFOXIRI plus bevacizumab as first-line treatment extended the median duration of overall survival (OS). In this study, we explored the effects of *UGT1A1* polymorphism on the outcomes of irinotecan dose escalation versus FOLFOXIRI plus bevacizumab in patients with *BRAF^V600E^*-mutant mCRC. *Materials and Methods*: We retrospectively reviewed the medical records of 25 patients who had received a diagnosis of *BRAF^V600E^*-mutant mCRC between October 2015 and August 2022. All patients underwent *UGT1A1* genotyping before receiving bevacizumab plus FOLFIRI. The primary end point was progression-free survival (PFS), and secondary endpoints were OS and adverse events (AEs). The two treatment arms were compared in terms of 6-month PFS and 12-month OS. *Results*: Over a median follow-up duration of 15.0 (interquartile range, 10.0–30.5) months, no significant differences were noted between the treatment arms in severe AEs (SAEs), 6-month PFS, or 12-month OS (all *p* < 0.05). Regarding AEs, the FOLFIRI plus bevacizumab regimen was associated with a lower incidence of anorexia than was the FOLFOXIRI plus bevacizumab regimen (*p* = 0.042). *Conclusions*: Our findings indicate that FOLFIRI plus bevacizumab with irinotecan dose escalation is an effective first-line treatment regimen for patients with *BRAF^V600E^*-mutant mCRC. This regimen leads to acceptable clinical outcomes with manageable AEs. However, the effects on survival and safety outcomes could only be speculated, and further studies are needed because of the sample size, the follow-up for the OS evaluation, and the non-uniformity in all the variables considered in the two groups.

## 1. Introduction

In Taiwan, colorectal cancer (CRC) is the most commonly diagnosed cancer (16,829 new cases in 2020) and the third leading cause of cancer-related mortality (6489 deaths in 2020) [1]. Approximately 25% of all patients with CRC receive a diagnosis of metastatic CRC (mCRC), and 25–30% of patients with an initial diagnosis of stage I–III CRC eventually develop mCRC [2,3,4,5]. With an improvement in the understanding of genetic drivers of tumor biology, CRC is evidently a complex and multifaceted disease with diverse molecular characteristics and distinct prognoses.

*BRAF*, a key gene affecting the mitogen-activated protein kinase pathway, mutated in 5–10% of all mCRC patients [6]. Oncogenic *BRAF* mutations lead to the constitutive activation of the aforementioned signaling pathway, promoting tumor proliferation and inhibiting apoptosis [7]. The *BRAF^V600E^* mutation, which accounts for approximately 90% of *BRAF* mutations, leads to the hyperactivation of the *BRAF* kinase activity in an *Ras*-independent manner. Patients with non-*BRAF^V600E^* mutations tend to be younger and are less likely to be female than those with *BRAF^V600E^* mutations. *BRAF^V600E^*-mutant CRCs are likely to affect the right side of the colon and present with distant lymph node and peritoneal metastases [8,9]. Seligmann et al. reported that the *BRAF^V600E^* mutation in patients with CRC is related to several clinical characteristics, including elder age, female sex, right-sided colon, poor differentiation, mucinous histology, and the presence of distant lymph nodes and peritoneal carcinomatosis [10]. The median duration of overall survival (OS) in patients with mutated-*BRAF^V600E^* mCRC ranges from 9.8 to 18.2 months, which is significantly inferior to that in patients with wild-type *BRAF* [7,11,12]. A key factor contributing to these poor clinical outcomes is reduced sensitivity to chemotherapy. Furthermore, patients with mutated-*BRAF^V600E^* mCRC are less likely to accept treatment following progression after first-line treatment due to rapid deterioration [10].

The optimal first-line treatment for *BRAF^V600E^*-mutant mCRC remains debatable. In the phase III TRIBE study, the median OS of patients with *BRAF^V600E^*-mutated mCRC treated with FOLFOXIRI plus bevacizumab was superior to that of patients treated with FOLFIRI plus bevacizumab [13]. However, treatment toxicity is still a concern, suggesting that the use of FOLFOXIRI as a first-line treatment option may be influenced by the performance status of patients with mCRC, as they might not be well suited for subsequent lines of therapy. Our previous investigations have revealed promising outcomes of irinotecan dose escalation, guided by uridine diphosphate glucuronosyltransferase 1A1 (*UGT1A1*) genotyping, in mCRC patients [14,15,16,17].

We previously demonstrated that first-line FOLFIRI plus bevacizumab and irinotecan dose escalation leads to favorable outcomes with tolerable adverse events (AEs) in patients with *BRAF*-mutated mCRC [18]. In the current study, we retrospectively analyzed real-world data and compared clinical efficacy and toxicity profiles between two first-line treatment approaches for patients with *BRAF^V600E^*-mutant mCRC: FOLFIRI plus bevacizumab with irinotecan dose escalation, guided by *UGT1A1* polymorphism, and FOLFOXIRI plus bevacizumab.

## 2. Materials and Methods

### 2.1. Study Design and Patients

This was a retrospective observational study and included patients who had received a confirmed diagnosis of synchronous or metachronous mCRC on the basis of histological evidence. All participants underwent standard screening tests, including *KRAS* (codon 12, 13, 59, 61, 117, and 146), *NRAS* (codon 12, 13, 59, 61, 117, and 146), *BRAF* (codon 600), and *UGT1A1* genotyping, and microsatellite instability (MSI) status assessment. Patients with *BRAF^V600E^* mutations were deemed eligible for inclusion in this study. We collected data regarding the patients’ demographic (age and sex), clinical (Eastern Cooperative Oncology Group (ECOG) performance status), and tumor (primary sites, synchronous or metachronous, *UGT1A1* status, MSI status, sites, and number of metastases) characteristics.

We retrospectively analyzed the medical records of patients with *BRAF^V600E^*-mutant mCRC who had received either FOLFIRI or FOLFOXIRI with bevacizumab as their first-line treatment. For patients in the FOLFIRI plus bevacizumab group, the treatment regimen followed a biweekly schedule and included bevacizumab 5 mg/kg + normal saline (N/S) 250 mL intravenous drip (IVD) for 2 h, followed by irinotecan + N/S 500 mL IVD for 4 h and leucovorin (200 mg/m^2^) + 5% glucose water (G/W) 250 mL + 5-FU (2800 mg/m^2^) + N/S 500 mL IVD for 42 h. Irinotecan was initiated at a dose of 180 mg/m^2^ in patients with *UGT1A1* TA6/TA6 and TA6/TA7, while it was initiated at a dose of 120 mg/m^2^ in patients with TA7/TA7; the dose was escalated on the basis of *UGT1A1* genotyping results and AEs. The estimated maximal dose of irinotecan was 260 mg/m^2^ for *UGT1A1 6TA/6TA*, 240 mg/m^2^ for *UGT1A1 6TA/7TA*, and 180 mg/m^2^ for *UGT1A1 7TA/7TA* [17]. A flowchart detailing the *UGT1A1* polymorphism dose escalation process is presented in Supplementary Materials Figure S1. Notably, patient consent and the occurrence of severe adverse events (SAEs) were considered during dose adjustment. For the patients in the FOLFOXIRI plus bevacizumab group, treatment was biweekly administered as follows: bevacizumab 5 mg/kg + N/S 250 mL IVD for 2 h, followed by irinotecan (165 mg/m^2^) + N/S 500 mL IVD for 4 h, oxaliplatin (85 mg/m^2^) + 5% G/W 250 mL IVD for 4 h, and leucovorin (200 mg/m^2^) + 5% G/W 250 mL + 5-FU (2800 mg/m^2^) + N/S 500 mL IVD for 42 h.

This study adhered to the principles outlined in the Declaration of Helsinki and the Clinical Practice guidelines. The research protocol was approved by the Institutional Review Board of Kaohsiung Medical University Hospital (KMUHIRB- E(I)-20200036). In this study, we compared clinical efficacy and toxicity profiles between the two aforementioned treatment groups.

### 2.2. Analysis of UGT1A1, RAS, and BRAF Polymorphisms and MSI Status

In order to analyze constitutional gene polymorphisms, DNA was first extracted from 4 mL of peripheral blood by using a PUREGENE^®^ DNA isolation kit (Gentra Systems, Minneapolis, MN, USA). Then, the extracted genomic DNA was subjected to direct sequencing to determine the sequence of the *UGT1A1* promoter region. Details of the genotyping procedures have been reported previously [14].

To genotype *RAS* gene, macrodissected paraffin-embedded samples were first placed in sterile tubes. After deparaffinization and rehydration, DNA was isolated using the PUREGENE^®^ DNA isolation kit. The primers used in this study were designed using the freely available primer design software Primer 3 (version 0.4.0) (http://bioinfo.ut.ee (accessed on 15 September 2023)). The sequences of the forward and reverse primers were 5′-TCATTATTTTTATTATAAGGCCTGCTGAA-3′ and 5′-CAAAGACTGGTCCTGCACCAGTA-3′, respectively. For polymerase chain reaction (PCR), the reaction volume was set at 40 μL. The PCR conditions for *KRAS* were as follows: initial denaturation 94.0 °C for 10 min; 35 cycles of denaturation at 94.0 °C for 30 s, annealing at 56.0 °C for 60 s, and primer extension at 72.0 °C for 30 s; and final extension at 72.0 °C for 7 min. The genotypes were verified through a fragment analysis of the PCR products by using automated capillary electrophoresis; for this, we used ABI PRISM 310 Genetic Analyzer and Genotyper (Applied Biosystems, Foster City, CA, USA).

For analysis of *BRAF* mutation, DNA was extracted from formalin-fixed, paraffin- embedded CRC tissue samples. This DNA was used for the clinical analysis of *BRAF* mutation direct sequencing. A comprehensive description of the genotyping procedures has been presented previously [18].

The deficient mismatch repair gene (dMMR) was determined through the immunohistochemical staining of CRC tissue specimens. The loss of one or more mismatch repair proteins (MLH-1, PMS-2, MSH-2, and MSH-6) was indicative of a dMMR or an MSI-high status [19].

### 2.3. Evaluation of Treatment Efficacy and Safety

After six cycles of treatment, we typically evaluated the tumor responses. The Response Evaluation Criteria in Solid Tumors version 1.1 was used to measure these responses [20]. The resultant AEs were closely monitored and graded during each treatment cycle according to the National Cancer Institute Common Terminology Criteria for Adverse Events version 4.3 (http://ctep.cancer.gov/reporting/ctc.html (accessed on 15 September 2023)). The definition of progression-free survival (PFS) was the interval between the date of enrollment and the first documented evidence of disease progression, regardless of treatment status. OS was defined as the interval between the date of enrollment and that of death or the latest follow-up. The achievements of completed responses (CRs) and partial responses (PRs) were collectively assessed in terms of objective response rates (ORRs). The disease-control rate (DCR) was estimated considering confirmed CR, PR, and stable disease. The PFS was defined as the primary end point, and the secondary end points were OS and AEs in this study.

### 2.4. Statistical Analysis

Patients who had successfully received the sixth cycle of treatment and were not lost to follow-up were enrolled in our analyses. Continuous variables are presented in terms of the mean ± standard deviation values, and categorical variables are presented in terms of the number and percentage values. All statistical analyses were performed using the SPSS (version 21; IBM Corporation, Armonk, NY, USA). The clinicopathological characteristics of the two groups were compared using Pearson’s chi-square test. Cox regression analysis was used to estimate the hazard ratios (HRs) for all independent variables in the model. PFS and OS were evaluated using the Kaplan–Meier method, and a log-rank test was used to compare time-to-event distributions. Statistical significance was set at *p* < 0.05.

## 3. Results

### 3.1. Population and Disposition

**Figure 1:**
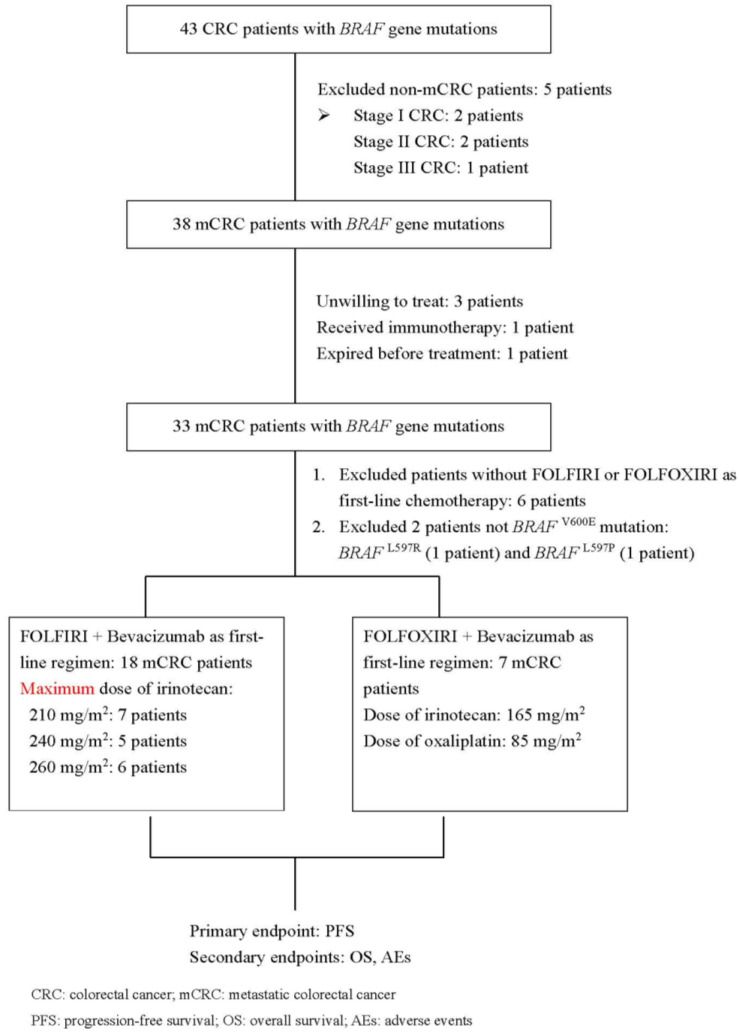
CONSORT diagram of the present study.

We retrospectively identified 43 patients who had received a diagnosis of the *BRAF* mutation between October 2015 and August 2022. Of them, five patients without mCRC were excluded from the analysis. Thus, 38 patients with *BRAF*-mutant mCRC were included. However, some ineligible patients within this group were subsequently excluded. Finally, 25 patients with *BRAF^V600E^*-mutant mCRC were included in the analysis. A flowchart depicting patient selection is presented in Figure 1. In the FOLFIRI plus bevacizumab group, approximately 37.5% (6/16) of the patients received irinotecan at a dose of 260 mg/m^2^. With the exception of MSI status, Table 1 shows that the baseline demographics and characteristics were similar between the two treatment groups.

Of the patients, 64.0% were aged <65 years and 64.0% were women. Only one patient (4.0%) had an ECOG status of 2. The two groups exhibited equal distributions of the sides affected by the primary lesion, with right-sided mCRC not being the predominant type. MSI status was not analyzed in 6 patients (24.0%); however, among the 19 patients who were subjected to MSI status analysis, 15 (78.9%) were found to have an MSI-low status and 4 (21.0%) were found to have an MSI-high status. No patient presented with the *UGT1A1 7TA/7TA* genotype. A total of 13 patients (52.0%) presented with peritoneal carcinomatosis alone or in combination with peritoneal metastases. In total, 17 patients (68.0%) presented with only one metastatic site. The final analysis of the database was last updated on August 31, 2023. As of the analysis cutoff point, the median follow-up duration was 15.0 (interquartile range, 10.0–30.5) months.

### 3.2. Treatment Efficacy

There were no significant differences to be observed between the FOLFIRI and FOLFOXIRI groups in terms of ORR (11.1% vs. 28.6%, respectively; *p* = 0.285; Table 2) or DCR (66.7% vs. 85.7%, respectively; *p* = 0.341; Table 2).

**Figure 2:**
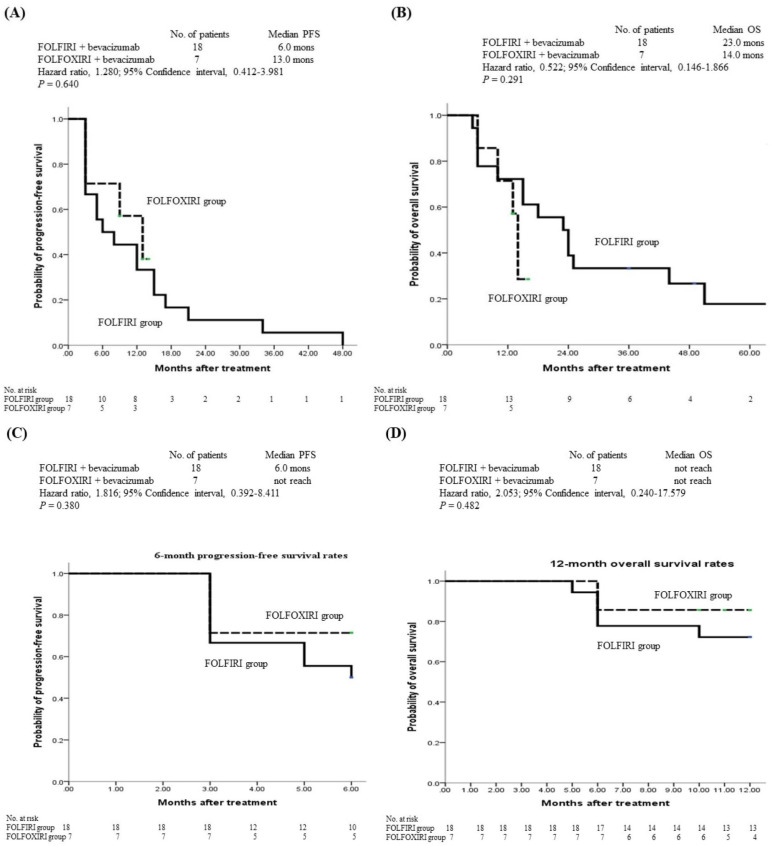
Kaplan–Meier survival curves for cumulative PFS and OS rates in the 25 included patients with *BRAF^V600E^*-mutant metastatic colorectal cancer. Differences in median PFS, median OS, 6-month PFS, and 12-month OS were analyzed using a log-rank test. The following results were obtained: (**A**) The median PFS did not vary significantly between the two treatment arms (*p* = 0.640). (**B**) The median OS did not significantly vary between the two treatment arms (*p* = 0.291). (**C**) Six-month PFS did not significantly vary between the two treatment arms (*p* = 0.380). (**D**) Twelve-month OS did not significantly vary between the two treatment arms (*p* = 0.482). PFS, progression-free survival; OS, overall survival.

In the FOLFIRI group, the median duration of PFS was 6.0 months and no patient was progression-free at the final follow-up. By contrast, the median duration PFS of the FOLFOXIRI group was 13.0 months and 4 (57.1%) of 7 patients were progression-free at the final follow-up (HR, 1.280; 95% CI, 0.412–3.981; *p* = 0.640; Figure 2A). The median duration of OS was 23.0 and 14.0 months in the FOLFIRI and FOLOXIRI groups, respectively, and 3 (16.7%) and 4 (57.1%) patients, respectively, remained alive at the final follow-up (HR, 0.522; 95% CI, 0.146–1.866; *p* = 0.291; Figure 2B).

We further compared the clinical efficacy between the two treatment regimens by evaluating 6-month PFS and 12-month OS. No significant between-group difference was noted in 6-month PFS (HR, 1.816; 95% CI, 0.392–8.411; *p* = 0.380; Figure 2C) or 12-month OS (HR, 2.053; 95% CI, 0.240–17.579; *p* = 0.482; Figure 2D). Regarding 6-month PFS, 50.0% (9/18) and 71.4% (5/7) of all patients were progression-free in the FOLFIRI and FOLFOXIRI groups, respectively. The median 6-month PFS was 6.0 months in the FOLFORI group; however, no patient achieved 6-month PFS in the FOLFOXIRI group. Similarly, an analysis of the 12-month OS revealed that no patient achieved the median 12-month OS in either treatment group and 77.8% (14/18) and 85.7% (6/7) of all patients remained alive in the FOLFIRI and FOLFOXIRI groups, respectively.

### 3.3. Treatment Safety

As shown in Table 3, the most common AEs were anemia (100.0% vs. 100.0%) and fatigue (100.0% vs. 100.0%) in both groups. The two groups significantly varied in terms of anorexia incidence, which was higher in the FOLFOXIRI group than in the FOLFIRI group (*p* = 0.042). Concerning grade III/IV AEs associated with escalated irinotecan doses, one hematologic event (5.6%) and two nonhematologic events (11.1%) were recorded in the FOLFIRI group. These events were exclusive to patients with the *UGT1A1 6TA/6TA* genotype who received an escalated irinotecan dose of 260 mg/m^2^. However, no significant between-group difference was noted in the occurrence of SAEs (*p* = 0.524 for hematologic SAEs and *p* = 0.358 for nonhematologic SAEs, Table 3).

A comparison of the two groups revealed no significant difference in the primary endpoint (PFS; *p* > 0.05) or the secondary endpoints (OS and AEs; *p* > 0.05).

## 4. Discussion

The importance of evaluating the efficacy of various treatment strategies has been emphasized in real-world populations based on the surge of interest in oncology research. These analyses help determine whether treatment standards established in tightly controlled clinical trials are truly applicable to a broader patient population. In the present study, we retrospectively reviewed the data of patients with *BRAF^V600E^*-mutant mCRC included from a single tertiary hospital (real-world setting) to analyze their clinical characteristics, treatment (FOLFIRI with irinotecan dose escalation plus bevacizumab) outcomes and toxicities, and oncological outcomes. The following key findings were obtained. A comparison of triplet chemotherapy with doublet chemotherapy with irinotecan escalation revealed no significant differences in median PFS, 6-month PFS, median OS, and 12-month OS. Trends toward more favorable ORRs and DCRs were observed for triplet chemotherapy; however, these results did not reach statistical significance. Doublet chemotherapy with irinotecan escalation had a less significant effect on appetite than did triplet chemotherapy. However, no significant differences were noted between these two regimens in the occurrence of SAEs.

CRC, similar to many other malignancies, is a heterogeneous disease; its subtypes are characterized by genetic alterations. For example, *BRAF* mutations occur in approximately 12% of all patients with mCRC, with recent estimates ranging from as low as 5% to as high as 21% [21,22,23,24,25]. Most of these mutations involve *V600E* substitution [11]. Several retrospective studies and meta-analyses have associated *BRAF*-mutant CRC with a particular phenotype. Older age at diagnosis and female sex are the main epidemiological features of patients with *BRAF*-mutant CRC [26]. The *BRAF* mutation is more commonly associated with pathological features such as proximal colon tumors, poor differentiation, mucinous histology, MSI, and large primary tumors [27,28,29]. The pattern of metastatic spread in *BRAF*-mutant CRC differs from that in *BRAF* wild-type tumors. *BRAF*-mutant CRC is more likely to present with peritoneal metastasis and less likely to have liver-limited or lung metastasis [30,31]. A recent study demonstrated that patients with mCRC with only peritoneal metastasis who had received first-line FOLFIRI plus bevacizumab, particularly with irinotecan dose escalation guided by *UGT1A1* polymorphism, achieved favorable outcomes similar to those of patients with mCRC with only liver or lung metastasis [32]. Moreover, in the present study, 64.0% of all patients were aged <65 years and 60.0% exhibited an MSI-low status. In our study, the prevalence of right-sided colon tumors was comparable to that of left-sided colon tumors and female patients were dominant. Furthermore, 52.0% of all included patients had peritoneal metastasis, either exclusively or in combination with other metastatic sites.

Irinotecan must be converted by a carboxylesterase to its active form, SN-38, which is then further metabolized by UGT. Marcuello et al. reported that patients with homozygous UGT1A1*28/*28 more frequently experience irinotecan-associated SAEs than do those with other genotypes [33]. Clinical presentations may vary across patients with UGT1A1*1/*28. These patients generally exhibit tolerance to the recommended initial dose of irinotecan (180 mg/m^2^) [34]. Conversely, patients with the homozygous UGT1A1*1/*1 genotype are more tolerant of higher irinotecan doses (as high as 260 mg/m^2^) [34]. According to the Pan-Asian adapted ESMO consensus guidelines, patients with favorable *UGT1A1* genotypes (homozygous wild *1/*1 and heterozygous *1/*28) can tolerate high-dose irinotecan but may experience significant toxicity [35]. UGT1A1*28 polymorphism has been recognized as a crucial determinant of irinotecan dose adjustment for the regulation of SAEs in patients with mCRC without compromising oncological outcomes [36]. Among patients with mCRC receiving FOLFIRI plus bevacizumab, those undergoing pretherapeutic *UGT1A1* genotyping and subsequent irinotecan dose adjustments have been demonstrated to achieve favorable outcomes without any substantial increase in AE incidence [14,15,16,17].

The standard first-line chemotherapeutic treatment for advanced *BRAF*-mutant mCRC involves a fluoropyrimidine-based cytotoxic regimen, which can include either irinotecan or oxaliplatin in combination with bevacizumab [37]. In the TRIBE study, the efficacy of FOLFIRI plus bevacizumab was compared with that of FOLFOXIRI plus bevacizumab as first-line therapy in a cohort of 508 patients with mCRC [13]. Among these patients, 28 had *BRAF^V600E^* mutations; 12 patients were in the FOLFIRI arm and 16 were in the FOLFOXIRI arm. In the TRIBE study, the median OS of patients with the *BRAF^V600E^* mutation who were treated with FOLFIRI plus bevacizumab was 10.7 months and that of those who were treated with FOLFOXIRI plus bevacizumab was 19.0 months (HR, 0.54; 95% CI, 0.24–1.20). Although an analysis suggested that patients with *BRAF^V600E^* mutations obtain similar benefits from FOLFOXIRI as do their *BRAF* wild-type counterparts compared with the benefits of FOLFIRI, this notion was not confirmed in the follow-up phase III TRIBE-2 trial, during which patients were randomized to receive FOLFOXIRI plus bevacizumab or FOLFOX plus bevacizumab followed by FOLFIRI plus bevacizumab [38]. In the present study, we found that the median OS was 23.0 months for patients with *BRAF^V600E^*-mutations who received FOLFIRI plus bevacizumab and 14.0 months for those who received FOLFOXIRI plus bevacizumab (HR, 0.522; 95% CI, 0.146–1.866; *p* = 0.291). In our study, the median OS for patients receiving FOLFIRI plus bevacizumab, inclusive of those with escalated irinotecan doses, was 23.0 months, and the median OS for patients receiving FOLFOXIRI plus bevacizumab in the TRIBE study was 19.0 months. We observed no significant difference in 6-month PFS or 12-month OS between these two treatment arms. A recent meta-analysis of five randomized trials comparing FOLFOXIRI plus bevacizumab to a doublet chemotherapy plus bevacizumab regimen revealed no prominent advantage of FOLFOXIRI plus bevacizumab [39]. Evidence for a clear benefit of triplet cytotoxic regimens over doublet chemotherapy in the first-line treatment of *BRAF^V600E^*-mutant mCRC appears to be insufficient.

In the TRIBE study, the ORR of patients with mCRC who were treated with FOLFOXIRI plus bevacizumab as first-line therapy was significantly higher than that of those who were treated with FOLFIRI plus bevacizumab. However, in our study, the increases in ORR and DCR for the FOLFOXIRI plus bevacizumab group compared with the FOLFIRI plus bevacizumab group did not reach statistical significance.

This study has some limitations that must be acknowledged. First, this study was retrospective in nature, had a small sample size, and was based on data from a single center. Second, UGT1A1*6 polymorphism is recognized as a potential predictor of irinotecan-related severe neutropenia. Nonetheless, this study did not include pretherapeutic UGT1A1*6 genotyping, in accordance with the Pan-Asian adapted ESMO consensus guidelines [35]. Third, the follow-up duration in this study was insufficient to evaluate long-term efficacy, particularly in terms of OS. Fourth, owing to the limited sample size in both groups, we could not perform a multivariate analysis with corrected estimates.

## 5. Conclusions

In conclusion, the present study provides real-world observational data from one single center and suggests that integrating higher-than-recommended doses of irinotecan into the FOLFIRI plus bevacizumab regimen for patients with *BRAF^V600E^*-mutant mCRC may be feasible. Oncological outcomes of FOLFIRI plus bevacizumab with irinotecan dose escalation were acceptable, with tolerable AEs and comparable 6-month PFS and 12-month OS. However, the non-inferiority of FOLFOXIRI plus bevacizumab vs. FOLFIRI plus bevacizumab with escalating iriontecan doses in patients with *BRAF*-mutated mCRC patients was not demonstrated with these preliminary data and the acceptability and AE tolerability have already been reported in our previous study [18]. Multicenter, prospective, randomized trials are warranted to validate our real-world observations.

## Figures and Tables

**Table 1:** Baseline characteristics of the 25 enrolled patients with *BRAF*-mutated mCRC receiving FOLFIRI plus bevacizumab or FOLFOXIRI plus bevacizumab as first-line regimen.

		FOLFIRI ^2^ Group	FOLFOXIRI ^3^ Group	
Baseline Data	*n* ^1^	*n* = 18	*n* = 7	*p* Value
		*n* (%)	*n* (%)	
Sex				0.158
Male	9	8 (44.4)	1 (14.3)	
Female	16	10 (55.6)	6 (85.7)	
Age (y/o ^4^)				0.455
Median (range)		59.5 (35.0–82.0)	58.0 (36.0–68.0)	
Age (y/o ^4^)				0.656
<65	16	12 (66.7)	4 (57.1)	
≥65	9	6 (33.3)	3 (49.2)	
ECOG PS ^5^				0.515
0	2	2 (11.1)	0 (0.0)	
1	22	15 (83.3)	7 (100.0)	
2	1	1 (5.6)	0 (0.0)	
Primary lesion site				0.568
Left-sided ^6^	12	8 (44.4)	4 (57.1)	
Right-sided ^7^	13	10 (55.6)	3 (49.2)	
Synchronous/Metachronous				0.943
Synchronous ^8^	14	10 (55.6)	4 (57.1)	
Metachronous ^9^	11	8 (44.4)	3 (49.2)	
MSI ^10^ status				0.039
MSI-H	4	4 (22.3)	0 (0.0)	
MSI-L	15	8 (44.4)	7 (100.0)	
NA ^11^	6	6 (33.3)	0 (0.0)	
*UGT1A1* genotyping				0.358
(6, 6)	23	16 (88.9)	7 (100.0)	
(6, 7)	2	2 (11.1)	0 (0.0)	
(7, 7)	0	0 (0.0)	0 (0.0)	
Metastatic sites				0.067
Liver	3	2 (11.1)	1 (14.3)	
Peritoneum	8	4 (22.2)	4 (57.1)	
Distant L.N. ^12^ metastasis	5	5 (27.8)	0 (0.0)	
Liver + lungs	2	2 (11.1)	0 (0.0)	
Liver + peritoneum	3	3 (16.7)	0 (0.0)	
Distant L.N. ^12^ + peritoneum	2	0 (0.0)	2 (28.6)	
Others	2	2 (11.1)	0 (0.0)	
No. of metastatic sites				0.819
1	17	12 (66.7)	5 (71.4)	
≥2	8	6 (33.3)	2 (28.6)	

^1^ *n*, number. ^2^ FOLFIRI, folinic acid + 5-FU + irinotecan. ^3^ FOLFOXIRI, folinic acid + 5-FU + oxaliplatin + irinotecan. ^4^ y/o, year-old. ^5^ ECOG PS, the Eastern Cooperative Oncology Group performance status. ^6^ Left-sided, descending colon + sigmoid colon + rectosigmoid colon + rectum. ^7^ Right-sided, cecum + ascending colon + transverse colon. ^8^ Synchronous, metastatic lesions occurred initially. ^9^ Metachronous, metastatic lesions occurred at least 6 months after resection of primary lesion. ^10^ MSI, microsatellite instability. ^11^ NA, non-analysis. ^12^ L.N., lymph node.

**Table 2:** The comparison of efficacy between the FOLFIRI plus bevacizumab group and the FOLFOXIRI plus bevacizumab group.

		FOLFIRI ^2^ Group	FOLFOXIRI ^3^ Group	
Efficacy	*n* ^1^	*n* = 18	*n* = 7	*p* Value
		N (%)	N (%)	
Response				0.446
Complete response (CR)	0	0 (0.0)	0 (0.0)	
Partial response (PR)	4	2 (11.1)	2 (28.6)	
Stable disease (SD)	14	10 (55.6)	4 (57.1)	
Progressive disease (PD)	7	6 (33.3)	1 (14.3)	
ORR ^4^				0.285
CR + PR	4	2 (11.1)	2 (28.6)	
SD + PD	21	16 (88.9)	5 (71.4)	
DCR ^5^				0.341
CR + PR + SD	18	12 (66.7)	6 (85.7)	
PD	7	6 (33.3)	1 (14.3)	

^1^ *n*, number. ^2^ FOLFIRI, folinic acid + 5-FU + irinotecan. ^3^ FOLFOXIRI, folinic acid + 5-FU + oxaliplatin + irinotecan. ^4^ ORR, objective response rates. ^5^ DCR, disease control rates.

**Table 3:** Common AEs ^1^ and severe AEs ^1^ of the FOLFIRI plus bevacizumab group and FOLFOXIRI plus bevacizumab group.

	FOLFIRI Group (*n* = 18)					FOLFOXIRI Group (*n* = 7)					
	*n*				*n* (%)	*n*				*n* (%)	
Grade	1	2	3	4	Overall Aes ^1^	1	2	3	4	Overall Aes ^1^	*p*-Value
Hematologic AE ^1^											
Anemia	11	6	1	0	18 (100.0)	4	3	0	0	7 (100.0)	-
Neutropenia	11	6	0	0	17 (94.4)	2	4	0	0	6 (85.7)	0.470
Thrombocytopenia	2	0	0	0	2 (11.1)	0	1	0	0	1 (14.3)	0.826
Non-hematologic AE ^1^											
Anorexia	6	2	0	0	8 (44.4)	2	4	0	0	6 (85.7)	0.042
Nausea	4	11	1	0	16 (88.9)	1	6	0	0	7 (100.0)	0.358
Vomiting	7	4	1	0	12 (66.7)	3	2	0	0	5 (71.4)	0.819
Diarrhea	1	0	0	0	1 (5.6)	0	0	0	0	0 (0.0)	0.524
Fatigue	1	17	0	0	18 (100.0)	1	6	0	0	7 (100.0)	-
Alopecia	1	0	-	-	1 (5.6)	0	0	-	-	0 (0.0)	0.524
Pruritus	1	0	0	0	1 (5.6)	0	0	0	0	0 (0.0)	0.524
Insomnia	1	0	0	0	1 (5.6)	0	0	0	0	0 (0.0)	0.524
Liver dysfunction	1	0	0	0	1 (5.6)	0	0	0	0	0 (0.0)	0.524
Acute renal injury	1	1	0	0	2 (11.1)	0	0	0	0	0 (0.0)	0.358
Severe Aes ^1^		Grade 3 & 4 *n* (%)					Grade 3 & 4 *n* (%)				*p*-value
Hematologic SAEs		1 (5.5)					0 (0.0)				
Anemia		1 (5.5)					0 (0.0)				0.524
Non-hematologic SAEs		2 (11.1)					0 (0.0)				
Nausea		1 (5.5)					0 (0.0)				
Vomiting		1 (5.5)					0 (0.0)				0.358

NOTE. Data are given as no. (%) except where otherwise noted. *p*-value was calculated by the Chi-square test (two-sided). ^1^ AEs: adverse events.

## Data Availability

The data and materials analyzed in the current study are available from the corresponding author on reasonable requests.

## Supplementary Materials

The following supporting information can be downloaded at: https://www.mdpi.com/article/10.3390/medicina59122108/s1, Figure S1: The escalated doses of irinotecan based on the *UGT1A1* polymorphisms.
