# Supplementary material for: Real-World Outcomes of First-Line FOLFIRI Plus Bevacizumab with Irinotecan Dose Escalation versus FOLFOXIRI Plus Bevacizumab in BRAFV600E-Mutant Metastatic Colorectal Cancer: The Preliminary Data from a Single-Center Observational Study
Source: Medicina (Kaunas). 2023 Dec 1;59(12):2108. doi: 10.3390/medicina59122108 (PMC10745094; doi:10.3390/medicina59122108)
Supplement: Supplementary file 1 [file medicina-59-02108-s001.zip › medicina-2641732-supplementary.pdf]

Supplementary Materials: Figure S1. The escalated doses of irinotecan based on the *UGT1A1* polymorphisms.

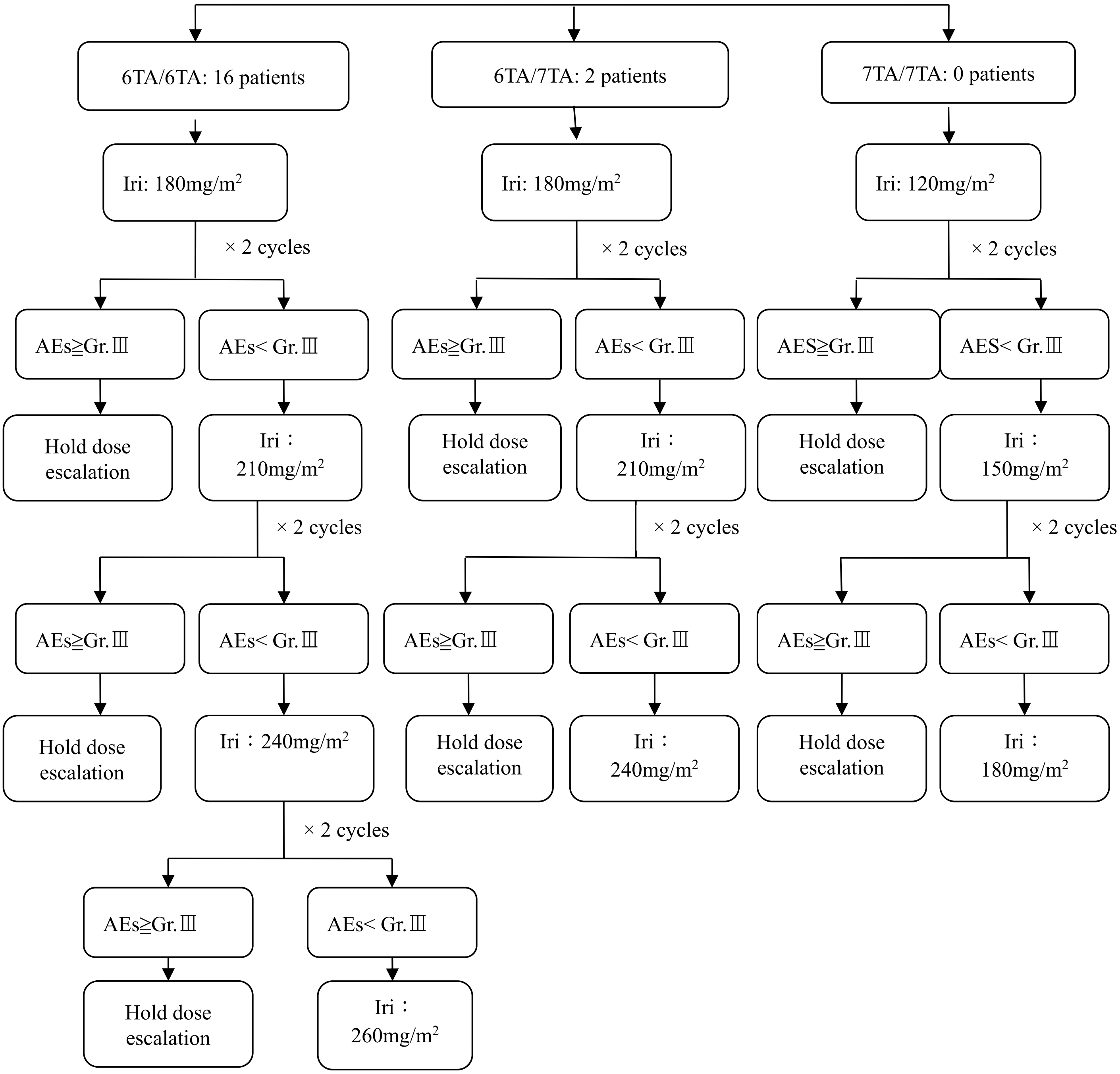

[1.]Among 16 patients with TA6/TA6, 6 patients with maximum dose of 260 mg/m^2; 5 patients with 240 mg/m^2 and 5 patients with 210 mg/m^2 respectively. [2.]Among 2 patients with TA6/TA7, 1 patient with 240 mg/m^2 and 1 patient with 210 mg/m^2, respectively. [3.]All of the three SAEs including one event of anemia, one event of nausea, and one event of vomiting. All of them occurred in the TA6/TA6 patients with maximum dose of 206 mg/m^2.
